# A Pilot Study Investigating Faecal Microbiota After Two Dietary Interventions in Children with Juvenile Idiopathic Arthritis

**DOI:** 10.1007/s00284-022-02899-1

**Published:** 2022-06-07

**Authors:** Lillemor Berntson, Anders Öman, Lars Engstrand, Johan Dicksved

**Affiliations:** 1 Department of Women’s and Children’s Health, Uppsala University, 75309 Uppsala, Sweden; 2 Centre for Translational Microbiome Research, (CTMR), Department of Microbiology, Tumor and Cell Biology, Karolinska Institutet, 17177 Stockholm, Sweden; 3 Department of Animal Nutrition and Management, Swedish University of Agricultural Sciences, Almas Allé 8, 75007 Uppsala, Sweden

## Abstract

There is evidence for an impact of the gut microbiota on the immune system, which has consequences for inflammatory diseases. Exclusive enteral nutrition (EEN) and the specific carbohydrate diet (SCD) have been demonstrated as effective anti-inflammatory treatments for children with Crohn’s disease. We have previously shown an anti-inflammatory effect from these nutritional treatments in children with juvenile idiopathic arthritis (JIA). The aim of this study was to investigate if improved clinical symptoms after EEN or SCD treatment in children with JIA could be linked to changes in faecal microbiota. We included sixteen patients with JIA (age 7–17 years), six for treatment with EEN and ten with SCD. EEN was given for 3–5 weeks and SCD for 4–5 weeks, with clinical and laboratory status assessed before and after treatment. Faecal samples were analysed for microbiota diversity and composition using 16S rRNA gene sequencing. Analyses of the faecal microbiota showed an effect on the overall composition with both interventions; the most striking result was a decreased relative abundance of the genus *Faecalibacterium* from EEN and of *Bifidobacterium* from SCD. The α-diversity decreased significantly from SCD (*P* = 0.04), but not from EEN (*P* = 0.22). Despite the study cohorts being small, both EEN and SCD were shown to impact the faecal microbiota. Future larger studies with a focus on metagenomics or metabolomics could possibly reveal a link and clarify the clinical effects of those nutritional regimens.

## Introduction

Juvenile idiopathic arthritis (JIA) is a group of chronic arthritides with childhood onset. There are seven JIA categories according to the current classification criteria of the International League of Associations for Rheumatology, indicating the heterogeneity of the disease [1]. The cause of the disease is considered multifactorial, but early life events that could influence microbiota are seen as contributing factors, i.e. antibiotics at early age, early weaning from breastfeeding and delivery by caesarean section [2–4]. The intestinal microbiota and its interactions with the mucosa and the gastrointestinal immune system are components that have recently been discussed as contributing in the disease progression [5]. Studies on patients with JIA have indicated an altered microbial composition in faecal samples, but the results are not consistent [6–10].

Since there is support for an imbalance of the gastrointestinal immune system in children with JIA, different non-medical interventions have been discussed. One hypothesis is that altering the composition and function of the intestinal microbiota, for example, through a dietary intervention, could have an influence on the gastrointestinal immune system [5]. There are very few studies in children aiming to decrease inflammatory activity through diet and the majority of those have focussed on inflammatory bowel disease (IBD), such as Crohn’s disease (CD) and ulcerative colitis (UC) [11].

Just like in JIA, an altered and less functional gut microbiota has been proposed to play a role in the pathogenesis of CD. A decrease in the abundance of the phylum *Firmicutes* and a reduction of *Faecalibacterium prausnitzii* have been observed in several studies of CD patients [12, 13].

In paediatric CD, use of exclusive enteral nutrition (EEN) has been shown to exert an anti-inflammatory and healing effect on the intestinal mucosa, leading to an improved nutritional status and a reduced need for corticosteroids [14]. EEN is a whole-protein polymeric formula, containing what is needed for complete nutrition. In children with CD treated with EEN, a combined positive health effect has been shown to correlate with a change in the pattern of short-chain fatty acids (SCFAs) in faecal samples, which were shifted in an anti-inflammatory direction by EEN [15]. The influence on the microbiota composition has shown very varying results, not consistent between studies, but a reduction in microbiota diversity has been a common finding [16]. In a previous study, it was shown that EEN treatment could improve clinical symptoms in children with JIA [17]. Seven patients were included and the EEN treatment had a varied but positive effect on all patients, with improvement in juvenile arthritis disease activity score (JADAS27), number of inflamed joints and minutes of morning stiffness. However, the previous study did not include any analyses of faecal samples.

A diet that has been shown to have beneficial effects on IBD in children is the specific carbohydrate diet (SCD). The diet induces clinical and biochemical remission in paediatric CD and UC [18, 19], but not complete healing of the intestinal mucosa [20]. SCD is a nutritionally balanced diet based on non-starchy vegetables, fruits, legumes, nuts, seeds, meats and fish. Dairy consumption is limited to well-fermented yoghurt and hard cheese, while cereal and grain-based products, as well as other foods containing large amounts of refined starch or added sugar, are excluded from the diet. This significantly reduces the intake of disaccharides typically abundant in a Western diet, such as sucrose and lactose, while increasing the intake of certain non-starch polysaccharides (dietary fibre) [18]. Furthermore, most processed food is excluded, as it may contain emulsifiers and additives, proven to have a negative impact on the intestinal mucosa in animal models [21].

In a new explorative study of 15 children with JIA, improvement of clinical scores and symptoms, showing an anti-inflammatory effect, was shown from SCD after four to five weeks on the diet in the majority of children [22]. SCD significantly decreased morning stiffness and pain, and improved physical function. Seven of the 15 children in that study had arthritis at inclusion; in five of the seven, the arthritis improved. Moreover, faecal SCFAs increased from SCD, with the most pronounced increase in butyrate levels.

The aim of the present study was to investigate if improved clinical symptoms after two different dietary interventions, EEN and SCD, in children with JIA, could be associated with changes in faecal microbiota composition.

## Material and Methods

### Study Design

Eligible for both nutritional interventions were patients < 18 years of age, diagnosed with JIA before 16 years of age, without suspicion of IBD, and with a normal faecal calprotectin level. Children could only be included if parents and children were strongly motivated to try a dietary intervention as a complementary treatment. Inclusion criteria for the intervention with EEN were also that patients were in an active phase of the disease, needing intra-articular corticosteroid injections, but they were in favour of starting EEN with a duration of at least 3 weeks, and wait 2 to 2.5 weeks with intra-articular corticosteroid injections to see any effect of the EEN. If the patient was on treatment with a DMARD, the treatment had to be in a stable phase and there should be no change in medical treatment during the study. Enteral nutrition was the sole nutritional source, given as either elemental, semi-elemental or polymeric formula, with total exclusion of a normal diet. Inclusion criteria for the intervention with SCD were that patients were on stable treatment—i.e. there had been no change in medical treatment during the three months preceding inclusion, except joint injections at the latest two months earlier—but not yet in remission, with no more than two active joints at inclusion and an erythrocyte sedimentation rate (ESR) of 30 mm at most. Before inclusion, an initial telephone appointment with a dietician was performed. The families received a recipe booklet, a list of permitted foodstuffs and a list of recommendations, before a visit to the paediatric rheumatology clinic was performed for inclusion. After the inclusion visit, families were instructed to get familiar with what food to eat and what to avoid during a period of two weeks at most. After this, the participants were to follow the SCD diet strictly for at least four weeks, with follow-up visits after two and four weeks on SCD. The families were also instructed to fill out a food diary during three days at the very end of the 4-week period. Children brought their own food to school. Six children with JIA were included in the dietary intervention with EEN for 3–5 weeks and ten children in the intervention with SCD for 4–5 weeks. The different time limits for inclusion criteria—3 and 4 weeks, respectively—were based on ethical and practical issues in treatment with EEN [17] and SCD [22]. Throughout both trials, the families had regular and close contact with and access to the dietician, 1–2 times per week, by email and telephone, and the physician, by email. In both trials, children had their first physical visit after two weeks on the dietary intervention.

**Fig. 1:**
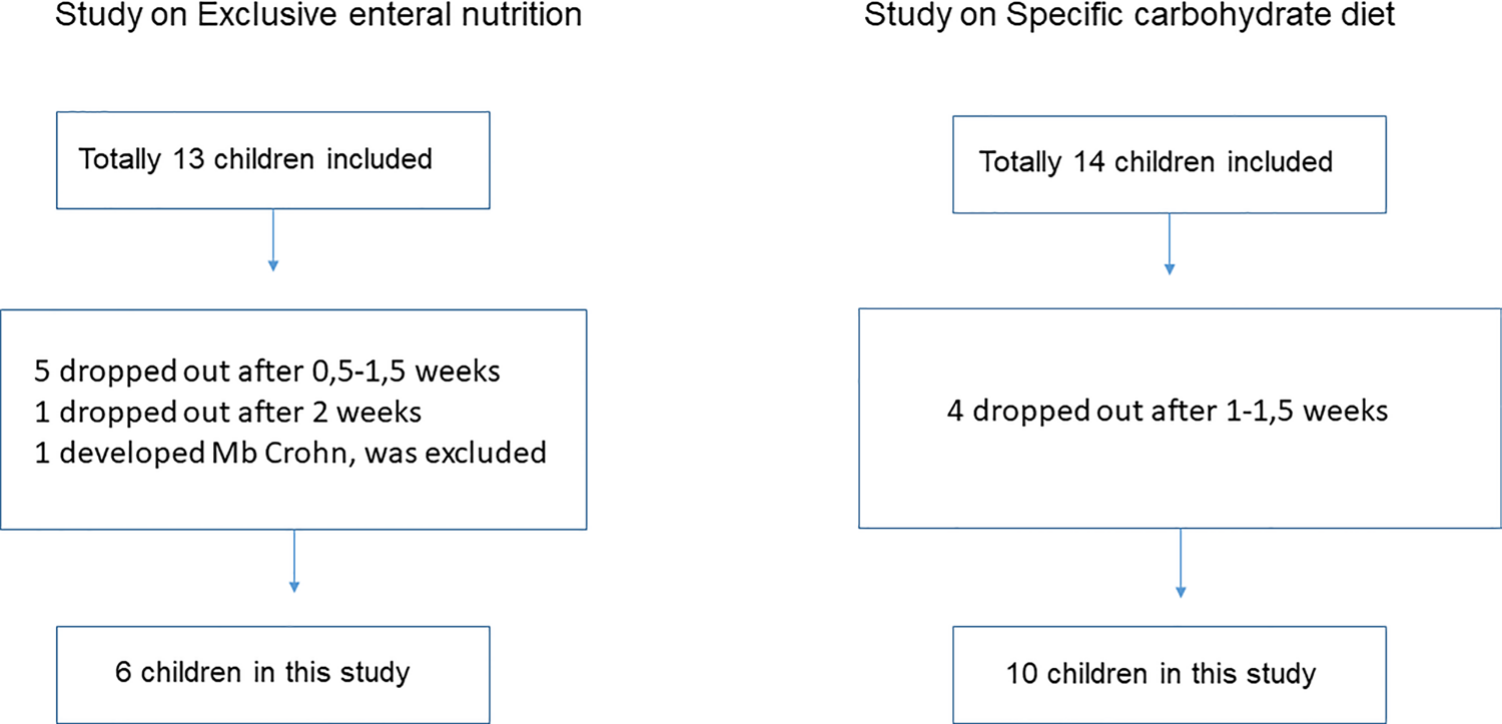
Flowchart of patients included in and excluded from the two studies

The number of children included in each study and the numbers of drop-outs are shown in a flowchart (Fig. 1). In the EEN study, five children stopped after 0.5–1.5 weeks of EEN because of poor compliance and one because of no effect after almost two weeks of EEN treatment and a desire to receive intra-articular corticosteroid injection at that time and discontinue the study. One further patient was excluded from this sub-study due to developing Crohn’s disease. Six patients had a clinical effect starting to be seen after 2–2.5 weeks of EEN and continued treatment for at least 4 weeks. In the SCD study, four families dropped out before two weeks of the study: in two, the child turned out not to be motivated enough, and in the other two, the parents were not motivated enough.

### Ethical Issues

The study on EEN was approved by the regional ethics committee in Uppsala County (Dnr 2012/378 and 2014/016). The study on SCD was approved by the regional ethics committee in Uppsala County (Dnr 2016/263, 2016/263/1, 2016/263/2) and by the Ethics Review Authority (Dnr 2020-01494). Written informed consent was obtained from the parents of children younger than 12 years and directly from children who were 12 years or older.

### Study Visits

Throughout the trial, the participants had regular contact with and access to the dietician, as well as a physician, through visits, email and telephone. Clinical and laboratory status were assessed before, during, and at the end of treatment periods. Disease activity was assessed based on JADAS27 (0–57). The JADAS27 comprises (1) the number of active joints (0–27), (2) patient global assessment visual analogue scale (0–10 cm), (3) physician global assessment visual analogue scale (0–10 cm) and (4) normalised ESR ((ESR (in mm/h) − 20)/10) to a scale 0–10 [23]. Arthritis was assessed at clinical examination, not using ultrasound at inclusion or follow-ups.

### Faecal Samples

Faecal samples were collected at inclusion and at follow-up after three to five weeks of EEN and after 2 and 4 weeks of SCD. In both groups, most faecal samples were collected at home and stored in + 6 °C degrees before being delivered to the hospital. At the hospital, samples were placed and stored in a − 70 °C freezer, at most 48 h after sampling.

### DNA Isolation

First, 1 mL of DNA/RNA shield (Zymo Research Corp, Irvine, CA) was added to 30–100 mg of frozen faecal sample. The samples were then subjected to bead beating with Matrix E (MP Biomedicals, Santa Ana, CA, USA) at 1,600 rpm using a 96 FastPrep shaker (MP Biomedicals, Santa Ana, CA, USA) for 1 min and then centrifuged for 4 min at 4400 rpm to remove beads from the solution. Then, 200 µL of the supernatant was incubated with 20 µL of lysozyme buffer (20 mM Tris–CL, 2 mM sodium-EDTA, lysozyme to 100 g/mL; Sigma-Aldrich, St. Louis, MO, USA) at 37 °C for 1 h at 1000 rpm and then at 80 °C for another 15 min and 250 rpm. Following this, samples were centrifuged for 5 min at 4400 rpm and 200 µL was transferred to a new plate, to eliminate larger particles. Next, 10 µL of proteinase K (from the Genomic DNA MagPrep kit, Zymo Research Corp, Irvine, CA, USA) was added, and the samples were then incubated at 55 °C for 30 min at 250 rpm. The samples were then cleaned through several washing and magnetic bead pelleting steps in accordance with the instructions of the manufacturer (Genomic DNA MagPrep kit, Zymo Research Corp, Irvine, CA, USA). Finally, genomic DNA was eluted from the magnetic beads with 70 µL of elution buffer (10 mM Tris–Cl, pH 8.5; Qiagen, Venlo, Netherlands).

### DNA Amplification and Sequencing

Indexed amplicons were generated in one PCR step using primers targeting the 341f-805r region of the 16S rRNA gene. The PCR mix contained 25 µL of Phusion Hot Start II High-Fidelity PCR MasterMix (ThermoFisher Scientific, Waltham, MA, USA), 1.5 µL DMSO and 4 µL of primers at 5 µM, plus 19.5 µL of DNA template at 1.03 ng/µL. Cycling conditions used an initial denaturation step of 80 s at 98 °C, 25 cycles of 10 s at 98 °C, 30 s at 53 °C and 30 s at 72 °C, and a final extension step of 2 min at 72 °C. PCR products were purified using Agencourt AMPure XP beads (Beckman Coulter AB, Stockholm, Sweden) and 80% ethanol and were eluted in PCR-grade water. Finished libraries were quantified using Invitrogen’s QuantIt fluorometric assay (Thermo Fischer Scientific, Waltham, Massachusetts, USA) before being pooled to equimolar amounts and sequenced in parallel to whole bacterial genomes in a MiSeq instrument (Illumina Inc, San Diego, CA, USA) using 2 × 300 bp reads and V3 chemistry.

### Sequencing Quality Control and Amplicon Sequencing Variant Picking

Raw sequencing reads were pre-processed with Cutadapt to remove adapter sequences, primer sequences, low-quality (< 15) 3′-bases and sequences containing any N [24]. The resulting reads were submitted to DADA2 [25] for merging, amplicon sequence variant (ASV) picking and taxonomy assignment based on the SILVA 128 database [26]. The final dataset was normalised to the sample with the lowest read count, 9907 sequences, to harmonise the number of sequence reads between samples. The sequence accession number for the sequence dataset is PRJNA778908.

### Statistical Analyses

Conventional descriptive statistics were used, with median and interquartile ranges given. For comparison of JADAS27 before and after nutritional intervention, the related samples Wilcoxon signed-rank test was used. *P* values of less than 0.05 on two-tailed tests were considered statistically significant. Analyses were carried out using the Statistical Package for Social Sciences version 26 (SPSS Inc., Chicago, IL, USA).

For all calculations and statistical analyses linked to the microbiota data, including analysis of diversity, we used the statistical software PAST, version 4.02 [27]. For assessment of microbial α-diversity, the Shannon diversity index, which encompasses both richness and evenness, was used and calculations were performed on the entire ASV matrix. To check for any impact of EEN and SCD on the microbial community composition, principal coordinate analysis (PCoA) plots were generated based on Bray–Curtis distances, and a similarity index with Bray–Curtis metrics was calculated to evaluate the change in microbiota composition from the intervention. The Bray–Curtis similarity is bounded between 0 and 1, where 1 means that two samples have the same composition (that is they share all the species as well as relative abundance of the species), and 0 means that two samples do not share any species. To test for changes in α-diversity and relative abundance for specific microbial taxa that were connected to the intervention, paired Wilcoxon signed-rank tests were used to compare samples from before and during treatment for EEN and SCD, respectively. The taxa analyses were performed on phylum and genus taxonomy data and included only taxa that were present in at least 50% of the samples in each intervention arm, and that were present with an average relative abundance > 0.1%. The Benjamini–Hochberg method was used to adjust for multiple comparisons, with a false discovery rate (FDR) of 5% [28]. Because of the explorative design, we also evaluated results not adjusted for multiple comparisons.

## Results

### Demographic Data

Figure 1 shows a flowchart of the children included in and excluded from each cohort. Demographic data are presented in Table 1. The improvements in JADAS27 for both interventions were significant (*P* = 0.03 for both). The medical treatment during the observation period was stable in all participants and is presented in Table 1. For the participants on the EEN intervention, results for inflammatory variables like ESR, physical function, as well as faecal calprotectin analysis results at inclusion for the same individuals (one excluded in this study because of developing CD) have been published previously [17]. Corresponding data regarding the study on treatment with SCD include all ten study participants in the SCD intervention [22].

**Table 1:** Demographic data in 16 patients with juvenile idiopathic arthritis (JIA) before and after two different nutritional interventions

	Patients on EEN *n* = 6	Patients on SCD *n* = 10
Female sex, *n* (%)	3/6 (50)	8/10 (80)
Age at disease onset, years, median (IQR)	9.9 (6.8–10.6)	12.2 (3.1–13.7)
Age at inclusion, years, median (IQR)	11.9 (9.6–16.4)	14.4 (11.4–16.8)
JADAS27 delta value, before/after treatment, median, IQR^a^	− 8.6 (− 13.9–(− 2.8))	− 3.2 (− 5.1–(− 1.2))

ILAR categories of JIA, course type	Number	Number
Oligoarticular persistent	1	4
Oligoarticular extended	0	1
Enthesitis-related arthritis	2	1
Polyarticular RF negative	3	3
Juvenile psoriatic arthritis	0	1
*Medical treatment during study*		
None	4	2
NSAID	0	2
Methotrexate	2	2
Biological agent	0	2
Biological agent + methotrexate	0	2

*IQR* interquartile range, *JADAS27* Juvenile Arthritis Disease Activity Score 27 (0–57), *ILAR* International League of Associations for Rheumatology, *RF* rheumatoid factor, *NSAID* non-steroid anti-inflammatory drug, *EEN* exclusive enteral nutrition, *SCD* specific carbohydrate diet

^a^Related samples Wilcoxon signed-rank test, *P* = 0.03 in EEN as well as in SCD

### Faecal Analyses

**Fig. 2:**
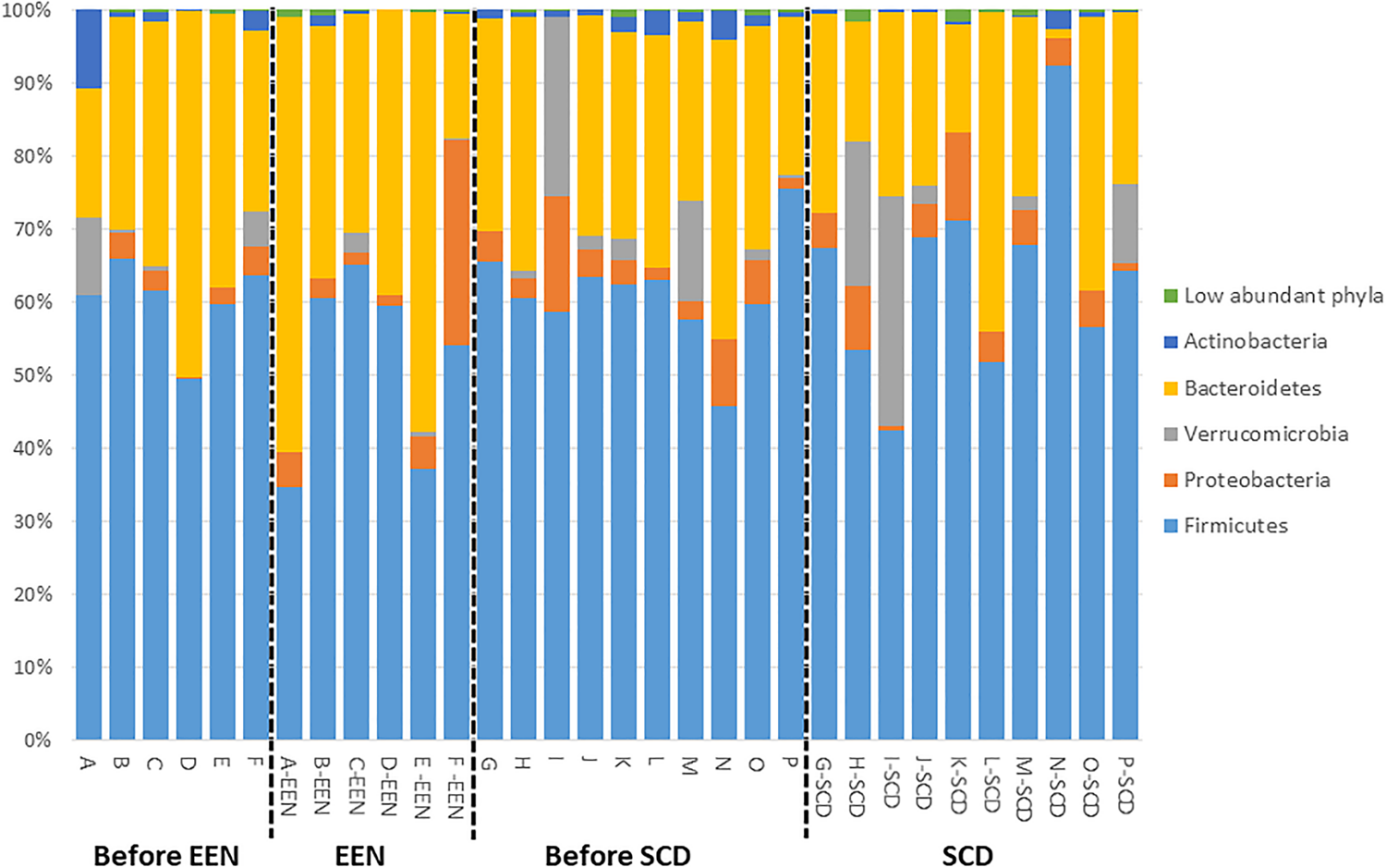
Relative abundance of bacterial phyla in faecal samples from children with juvenile idiopathic arthritis (JIA) collected before and during diet interventions. Six children with JIA (A–F) before and during exclusive enteral nutrition (EEN), and ten children with JIA (G–P) before and during specific carbohydrate diet (SCD)

**Fig. 3:**
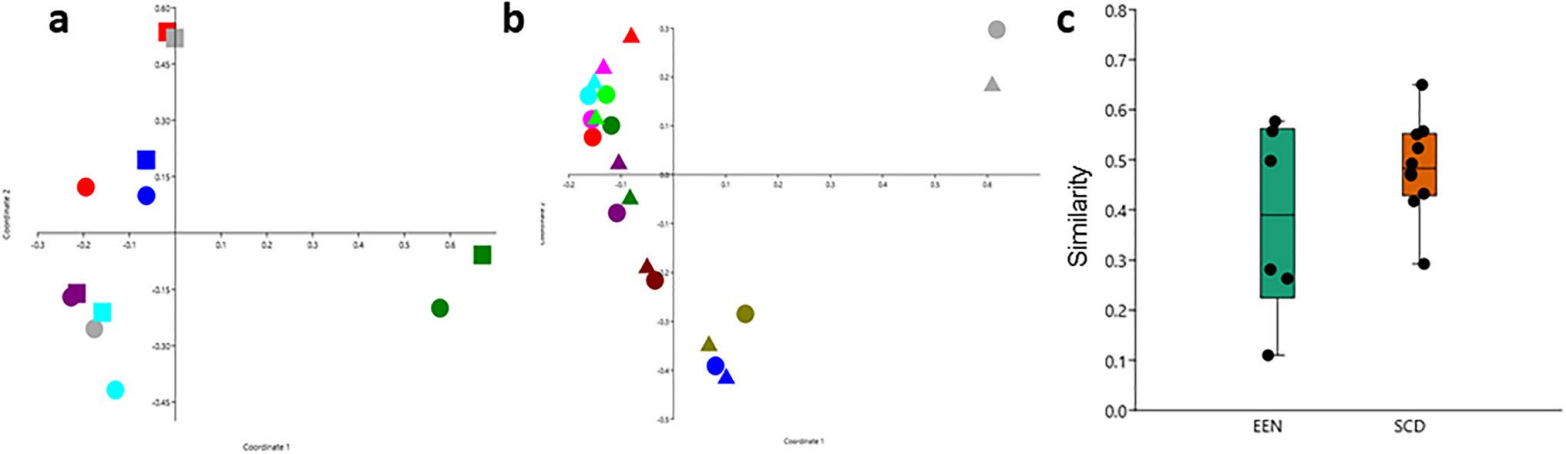
Participants are not the same in **a** and **b**. The colouring of dots has been made separately for **a** and **b**. **a** Principal coordinates analyses of community composition of faecal microbiota in samples from children with juvenile idiopathic arthritis (JIA) before and during diet interventions with exclusive enteral nutrition (EEN). Different colours represent different study participants. Dots represent samples collected before and squares represent samples collected during the EEN intervention. **b** Principal coordinates analyses of community composition of faecal microbiota in samples from children with juvenile idiopathic arthritis (JIA) before and during diet interventions with specific carbohydrate diet (SCD). Different colours represent different study participants. Dots represent samples collected before and triangles represent samples collected during the SCD intervention. **c** Boxplots and jitter plots showing the distributions of similarity indices based on Bray–Curtis metrics. Each dot represents the similarity in microbiota composition, in each individual, between the faecal sample collected before and the faecal sample collected during the diet intervention. A high value on the y-axis represents a high similarity between the variable composition in two samples. Boxplots show the medians and interquartile ranges whereas the whiskers represent min and max values

**Fig. 4:**
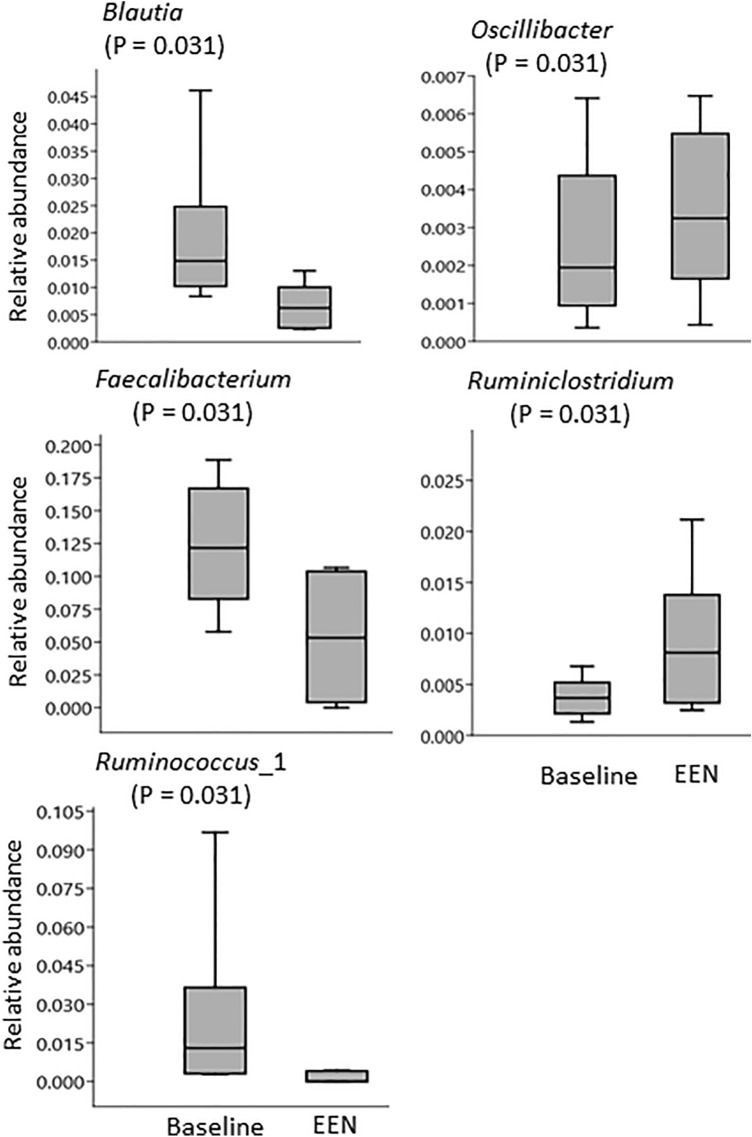
Boxplots for relative abundance of bacterial taxa at baseline, before EEN intervention and during EEN in six children with JIA. Selected taxa with a *P* value < 0.05 before correction for multiple analyses

**Fig. 5:**
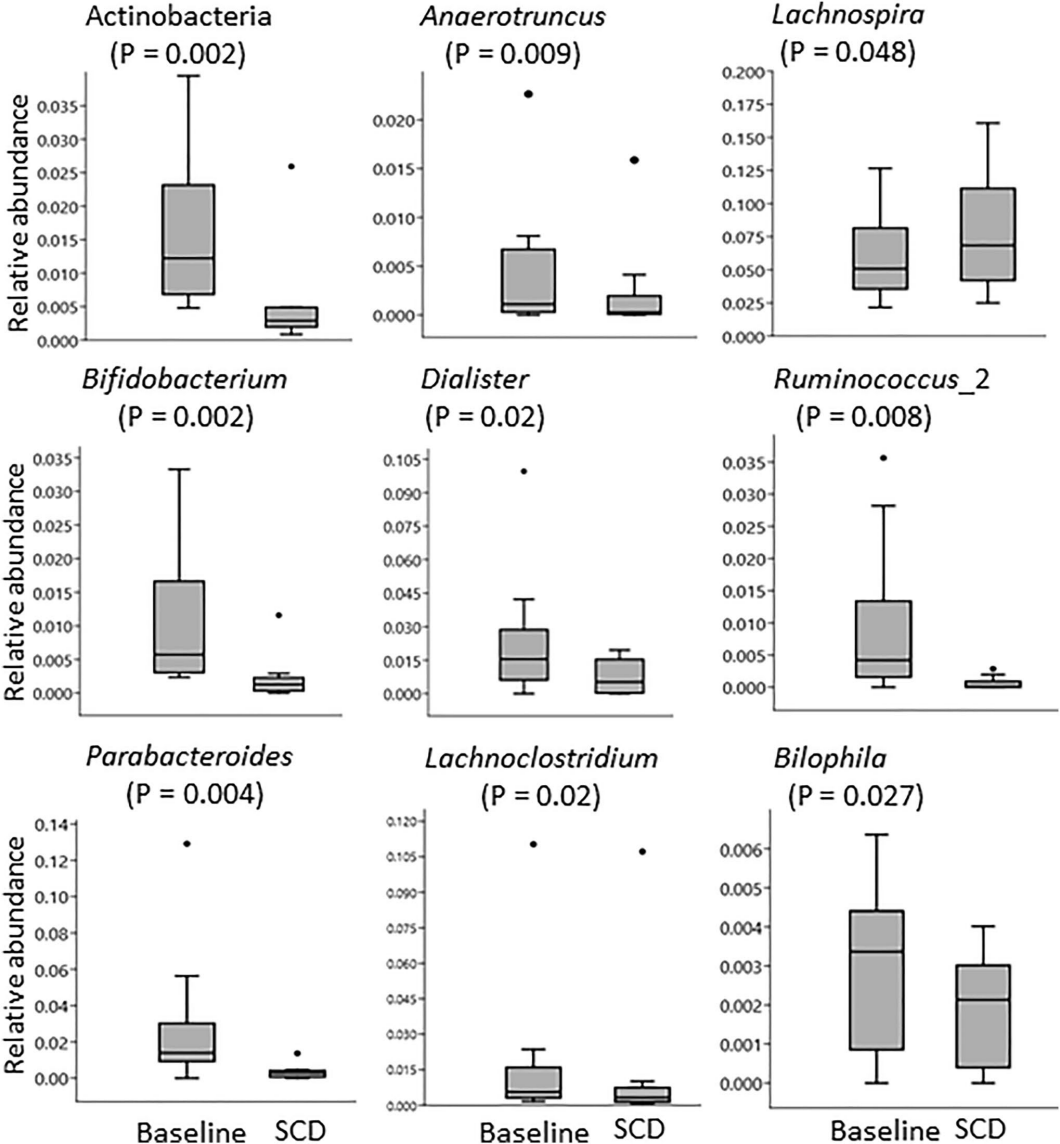
Boxplots for relative abundance of bacterial taxa at baseline, before SCD intervention and during SCD in ten children with JIA. Selected taxa with a *P* value < 0.05 before correction for multiple analyses

The analysis of microbiota composition revealed that samples were dominated by the *Firmicutes* and *Bacteroidetes* phylum, but with some differences in proportions between individual samples (Fig. 2). The paired analysis of Shannon’s diversity index before and during the intervention showed that the α-diversity decreased significantly for SCD (mean ± SD before SCD: 4.11 ± 0.49; during SCD: 3.87 ± 0.53, *P* = 0.04) but not for EEN (mean ± SD before EEN: 3.94 ± 0.78; during EEN: 3.74 ± 0.50, *P* = 0.22). The paired samples, before and during treatment, presented in PCoA plots, clustered by individual rather than by diet (Fig. 3a, b). Three children in the EEN group had a pronounced shift in community composition during treatment with EEN, and all children treated with EEN showed the same trend, with a shift in community composition in a similar direction. The microbial response to the SCD treatment varied more between participants and was without a clear trend (Fig. 3b). There was no significant difference between the two interventions when comparing similarity scores before and after the interventions, but three of the participants on the EEN intervention and one participant on the SCD diet showed a similarity score below 0.3, indicating a larger shift in the microbiota composition (Fig. 3c). In order to identify specific microbial taxa that were affected by the interventions, univariate paired tests were performed, comparing the relative abundance of individual taxa before and after EEN and SCD treatments. These tests revealed that certain genera were affected by EEN (Fig. 4) and SCD treatment (Fig. 5). However, after FDR adjustment of the statistical tests, the changes were not significant. At the phylum level, *Actinobacteria* decreased from the SCD treatment (*P* = 0.002), and that decrease was significant also after FDR adjustment (Fig. 5).

## Discussion

This pilot study evaluated the effects on the microbiota of two dietary interventions that contributed, to varying degrees, to an improved clinical picture in sixteen patients with JIA, six treated with EEN and ten with SCD. The analyses of faecal samples from before and during treatment revealed an impact on the overall composition of the faecal microbiota and a numerical decrease in α-diversity for both interventions, with a significant decrease from SCD. Medical treatment was stable during the interventions and a previous study has shown no significant influence on faecal microbiota from treatment with methotrexate and/or etanercept in children with JIA [29].

Past experiences of EEN treatment in children have mainly been gathered in studies of patients with CD. The exact mechanism of the anti-inflammatory effect seen with EEN therapy remains unknown, but one main theory is that EEN changes the gut microbiota and achieves mucosal healing by suppressing certain bacterial species and changing their metabolism [30, 31]. However, studies in children with CD treated with EEN have shown diverging results regarding impact on specific bacterial taxa in faecal samples. The most frequently reported effect from EEN has been a reduction in microbiota diversity [16]. This study also showed a lower numerical diversity on EEN treatment, although it did not reach statistical significance. The individual response in microbiota community composition to EEN treatment differed between patients, but in three of the six children, there were obvious shifts in the community composition. All six children had similar trends. Comparisons of relative abundance of taxa showed significant changes in the abundance of five genera before adjustment for multiple comparisons. An interesting finding was the change in relative abundance of *Faecalibacterium,* with lower relative abundances after EEN treatment compared with before treatment. *F. prausnitzii* is one of the dominant members of the *Firmicutes* phylum in faecal samples of healthy individuals [32] and is considered to be of particular importance in the microbe–host immune regulation, with studies demonstrating that it has anti-inflammatory properties [33]. The abundance of *F. prausnitzii* in faeces has been found to be lower in children with CD as compared with healthy controls [34], and also in one category of JIA: enthesitis-related arthritis [7, 35]. The decrease in *Faecalibacterium* from EEN in the present study is thus noteworthy, since both children with CD and children with JIA seem to have an anti-inflammatory effect from EEN [14, 17], despite the decreased abundance of *F. prausnitzii*. However, the decreased level of *Faecalibacterium* is not unexpected, since soluble fibres are lacking in EEN treatment. This finding is also in line with the results from at least three other studies, which all showed a decrease of the relative abundance of *Faecalibacterium* after EEN treatment in children with CD [30, 31, 36]. Further, the current study showed a decreased relative abundance of the genus *Blautia* as well as of one *Ruminococcus* genus, *Ruminocuccus* 2, from EEN treatment, which could be due to the lack of fibre in the EEN diet [31].

One previous study evaluated the impact of SCD on the microbiota in nine children with IBD and the main result was a decrease in the phylum *Proteobacteria* [19]. Also in the present study, there were changes in the microbiota during SCD treatment, with a significantly lower diversity in faecal samples collected during SCD treatment, and a decrease in the relative abundance of the phylum *Actinobacteria,* which was consistent also after correction for multiple analyses. The main genus within the *Actinobacteria* phylum is *Bifidobacterium*, which was present in a lower relative abundance after the SCD treatment. *Bifidobacterium* is commonly considered to be a beneficial and health-promoting bacterium in the gut [37]. *Bifidobacterium* has been proposed as a possible probiotic treatment for inflammatory diseases, and a study on adult patients with UC showed improved clinical symptoms during probiotic treatment with *Bifidobacterium longum* [38]. However, an increased abundance of *Bifidobacterium* has been shown in children with the enthesitis-related arthritis, one of the categories of JIA, and there is also a study on patients with UC showing a higher abundance of *Bifidobacterium* in patients compared with healthy subjects [7, 39]. The amount of *Bifidobacterium* in the gut is influenced by intake of milk products [40] and whole grains, especially rye [41, 42]. This could explain the lower levels in this study, since milk products and grains are largely excluded in SCD. In the pilot study [22], presenting clinical data and faecal SCFAs for the first 15 children in the SCD project, the levels of the SCFA butyrate increased significantly. The results from analyses of different taxa cannot explain the increase in butyrate, but it is known that many bacteria are involved in butyrate production. The genus *Lachnospira*, which increased from SCD, has been shown to increase from a diet rich in vegetables [43]. *Lachnospira* belongs to the family *Lachnospiraceae* [44], but the present study showed no change in the abundance of *Lachnospiraceae* from SCD. However, this family has been shown with a lower abundance in patients with enthesitis-related arthritis compared with healthy controls, in one dataset [7].

It is hard to find any similarities in the impact of SCD on the faecal microbiota between the present study and the study by Suskind et al. in children with CD [19]. Studies on EEN and SCD treatment in children with IBD are not entirely comparable with studies on children with JIA, since they are performed on children with different diseases, who might experience differing impact on microbiota. The results of diet interventions on microbiota composition are likely to depend on the baseline composition, which are expected to differ between children with IBD and children with JIA.

This study has several limitations. The study groups were small and included only six and ten patients, respectively. Therefore, there is a risk that differences caused by the treatment were not detected and all conclusions should be drawn with caution. There was no possibility to include control patients, since it would be unethical and far too difficult to motivate parents and healthy children to have the children on either of the strict nutritional regimens for weeks. It is possible that the participants in both studies who dropped out at 1.5–2.0 weeks of treatment had a feeling that the treatment was not successful. This could create a bias, even if a positive effect could started to be shown at 2–2.5 weeks at the earliest. Not least, knowledge is lacking regarding the influence on clinical status from a specific microbiota composition.

## Conclusion

In summary, in this study, treatment with both EEN and SCD affected the intestinal microbiota, but to some extent in a contradictory way, with a decreased relative abundance of certain taxa that are considered to be beneficial faecal bacteria, and a reduction in diversity during SCD. These findings in children with JIA need to be confirmed in larger cohorts, but one could speculate that the results of a shift in those taxa are not negative in the total setting of the complex bacterial composition. It is probable that the positive clinical effects of EEN and SCD are linked to an effect on the gut microbiota, but perhaps to changes in the function of microbiota rather than to changes in the abundance of specific taxa. Possibly, analysis of gut microbiota using metagenomics would reveal changes in function of gut microbiota from EEN or SCD.

## Data Availability

The datasets generated and/or analysed during the current study are not publicly available for ethical reasons, as well as privacy reasons, but are available from the author on reasonable request.

## Abbreviations

EEN: Exclusive enteral nutrition;
JIA: Juvenile idiopathic arthritis
SCD: Specific carbohydrate diet
SCFA: Short-chain fatty acids
IBD: Inflammatory bowel disease
CD: Crohn’s disease
UC: Ulcerative colitis
JADAS27: Juvenile arthritis disease activity score 27
PCoA: Principal coordinate analysis
ESR: Erythrocyte sedimentation rate
FDR: False discovery rate
RF: Rheumatoid factor
ILAR: International League of Associations for Rheumatology
NSAID: Non steroid anti-inflammatory drug
IQR: Interquartile range
